# Analysis of factors related to the morphological evolution of Lingnan export mugs in the 18th-20th centuries in the context of one belt and one road

**DOI:** 10.1371/journal.pone.0304104

**Published:** 2024-08-16

**Authors:** Jinghui Ao, Zilin Xu, Weicong Li, Miao Zhao, Qian Xie, Shanshan Ji

**Affiliations:** 1 School of Art and Design, Guangdong University of Finance and Economics, Guangzhou, China; 2 Faculty of Built Environment and Surveying, Universiti Teknologi Malaysia, Skudai, Johor Bahru, Johor, Malaysia; 3 School of Architecture & Applied Arts, Guangzhou Academy of Fine Arts, Guangzhou, China; 4 School of Digital Creative Design, Guangdong Nanhua Vocational College of Industry and Commerce, Guangzhou, China; University of Limpopo, SOUTH AFRICA

## Abstract

As a significant trade item on the ancient Silk Road, the evolution of mug shapes represents a confluence of Eastern and Western economic history and cultural-artistic exchanges, also reflecting the flourishing export culture of Guangzhou. This paper analyzes the functional and social factors influencing the morphological changes of Lingnan mugs from 1616 to 1949 from the perspective of quantitative typological analysis. The overall design trend of these mugs transitioned from complex to simple, enhancing user comfort, while variations in mug scale reflect the diversity of consumer classes and regional drinking cultures. Among the 30 mugs analyzed, the average capacity was 356ml, with a range of 1588ml. Common shapes included cylindrical bodies and ear-shaped handles. Morphologically, the belly of the mugs transformed from arc-barrel bodies (emphasizing heat retention) to bulbous bodies, and eventually to cylindrical bodies (combining heat retention, practicality, and economy), with handles also showing signs of East-West integration. The analysis of the mug body’ s inclination, with handle-side junction angles ranging from 34° to 53° and wall-side junction angles from 50° to 90°, indicates that these features are associated with stability in placement, aesthetic design, and practicality in liquid containment. These morphological evolutions reflect genuine responses to market demands and advancements in production technology, manifesting as products of market orientation and societal needs. By measuring changes in morphology, scale, volume, and external contour curves, this paper addresses how social factors shape material morphology in an academic context.

## 1 Introduction

Research on cultural heritage has expanded from focusing solely on the physical artifacts to encompassing aspects of cultural dissemination, forming two primary dimensions of interest: the ’social layer,’ focused on by sociologists and historians, and the ’craftsmanship layer,’ of interest to conservators. The former explores the interactivity between social structures and material culture [1], while the latter concentrates on the analysis of technical evolution, largely disconnecting from the cross-regional framework of cultural gene theory [2]. Casolino et al. [3] attempt to bridge ’social dynamics’ and ’technological innovation,’ seeking to find their interplay through macro sociological theories and micro-technological analyses, thus comprehensively explaining the evolution of material morphology. Drawing on constructivist and functionalist theories, the social construction of technology theory reveals the social dependency of technological choices and underscores how social groups embody their values through technological practices, thereby shaping the evolution of material culture [4].

Since the release of the United Nations’ "Convention Concerning the Protection of the World Cultural and Natural Heritage" [5] in 1972 and the "Convention on the Protection and Promotion of the Diversity of Cultural Expressions" [6] in 2004, the restoration and protection of cultural heritage have become a global consensus. The Guangdong government has introduced a series of policies to strengthen the restoration and protection of tangible cultural heritage, gradually establishing a scientific policy protection system [7,8]. At the beginning of the 20th century, the Italian conservator Brandi [9] first proposed contemporary theories on the conservation and restoration of cultural heritage, asserting that restoration must be based on a deep understanding of the artifacts, to reconstruct the inherent unity of the artworks. Addison [10] championed this theory and introduced the cutting-edge concept of "digital cultural heritage," providing a theoretical basis for the study of the relationship between tangible culture and digital technology, which has garnered widespread attention. Practically, Moisieiev [11] used morphological studies to classify unearthed porcelain from the Greek Crimea heritage area, clearly demonstrating the development and evolution of medieval porcelain production processes. Pawlowicz and Downum et al. [12] incorporated natural evolution into typological methods, proving that machine deep learning can assist in reassembling similar porcelain fragments, providing clues for understanding the production techniques and morphological evolution of ancient porcelain.

The cultural formation of export porcelain can be traced back to the late sixteenth century, reaching its peak in the mid-Qing dynasty, and establishing its fundamental modern design during the Republic of China period [13]. As documented by the Chinese Maritime Customs Service in the "Customs’ Gazette" [14], from 1602–1695, up to 20 million pieces of export porcelain were shipped from Guangzhou port to Europe [15]. However, Guangzhou’s export culture appears to be losing its uniqueness in the face of cultural globalization [16]. Local policies in Guangdong seem to favor economic value over cultural heritage preservation, thereby marginalizing cultural transmission and significance to some extent [17]. As national identity and cultural heritage become priorities, a plethora of culturally-inspired derivative products have emerged, although current designs are criticized for being overly commercialized and lacking in innovation [18].

Historically, export porcelain served not merely as a conduit for economic exchange but also as a vehicle for cultural interaction [19]. In contemporary times, it is increasingly viewed as a significant testament to historical, political, and cultural geography [20]. Most studies discuss the crafting and cultural transmission of excavated porcelain.

Lu [21], Han and Li [22], Meng and Zhou [23], and Liu [24] have noted that traditional art observation methods in analyzing the material morphology and cultural context of porcelain possess inherent subjective limitations. They have adopted typological morphology from archaeology to categorize and compare the morphology, craftsmanship, and traditional patterns of porcelain. For example, the morphological difference method was applied to analyze the morphology and functionality of Yuan dynasty high-footed porcelain cups [22], and quantitative typological analysis was used for the morphology and handling comfort of Tang dynasty porcelain ewers [23]. These studies emphasize the analysis of morphology and functionality. On a cultural level, Anastasi et al. [25] discovered that porcelain fragments found underwater might have been cargo transported early between North Tunisia and Sicily, providing evidence of early trade in the Mediterranean region. Li [26] noted that the most common decorative element in export porcelain was floral patterns, which with their fresh, delicate, and fine curves closely resemble the European Rococo style and have significantly influenced the cultures along the Belt and Road route. Chen and Ye [27] found that porcelain from the Philippines was dignified and sturdy, with a clear and bright celadon glaze, fine crackles on the surface, and typical Chinese porcelain decorative elements such as intertwined flower and lotus petal patterns, providing evidence of early maritime cultural exchanges between China and the Philippines.

The discussed studies highlight two core themes: 1) how interdisciplinary approaches can more effectively interpret the role of export porcelain in global cultural exchanges; and 2) how export porcelain reflects the socio-economic structure and cultural shifts of its production regions. External factors have been acknowledged as crucial in shaping the decorative art of export porcelain, prompting a shift towards trans-regional cultural studies. Few Chinese scholars have explored the cultural logic behind the formation of export porcelain decorative arts from a broad social perspective.

The objective of this study is to employ quantitative typological analysis to identify the factors influencing the evolution of mug morphology. By analyzing the morphology of Lingnan mugs from the 18th to the 20th century, this paper examines how the evolution of their forms has responded to societal needs and developments. Initially, samples and data from mugs were collected, and based on the attributes of these vessels, categorization and modeling were conducted. Subsequently, by typifying the morphology, this study uncovers the stylistic characteristics of mug evolution over different periods and analyzes the factors influencing these changes against the backdrop of their respective eras.

## 2 Methodology

### 2.1 Quantitative typological analysis

Quantitative typological analysis, a method that integrates statistical and computational techniques, is applied to analyze the morphological features of archaeological artifacts [28]. This approach not only facilitates precise categorization of the subjects under study but also highlights the distinctions across different cultural phases. Crucially, it reveals paths of cultural evolution, digitizing and visualizing cultural trends, thus enhancing the efficiency of archaeological research by providing a cost-effective and efficient framework that optimizes processes and conserves resources.

This methodology has found applications across fields such as archaeology, sociology, and design. Utilizing morphological studies, Kalilu et al. [29] categorized unearthed African textiles into 11 subcategories and two main categories of hand-made and machine-made laces, establishing a localized systematic classification framework. By incorporating deep learning, Bustos-Perez et al. [30] applied typological measurements to analyze the shape variations of Backed flakes from the Old Stone Age, providing a case reference for the digital application of heritage artifacts. Combining morphology and typology, Osipova et al. [31] analyzed the similarities and differences in the shapes of unearthed handaxes, offering archaeological evidence of cross-regional cultural exchanges between the Aral Sea area and the Mugalzhar Mountains.

The steps in applying quantitative typological analysis are as follows [19]: 1) Collecting primary data from digital collections of various museums to ensure completeness and reliability of samples; 2) Using Rhino software for three-dimensional modeling and making physical restorations based on rotatable observation 3D images from digital collections. Multi-point calibration is conducted based on measurements such as radius, aperture, and base diameter to obtain precise 3D models; 3) Measuring the morphology of mugs in Rhino to supplement missing data from some samples; 4) Aligning all models in Rhino to a unified elevation view and importing the images into AutoCAD 2022 to create a database. The mug body and handle morphologies are then categorized, coded, and compared based on the structural and attribute characteristics of the objects under study; 5) Calculating and measuring related morphological data in AutoCAD software and integrating the contour curves belonging to the same codes into a unified coordinate system to construct a chart that compares their proportions, scales, and differences; 6) Drawing a chart of the periodic morphological evolution of mugs and analyzing the underlying social driving factors.

### 2.3 Data sources and processing

**Fig 1:**
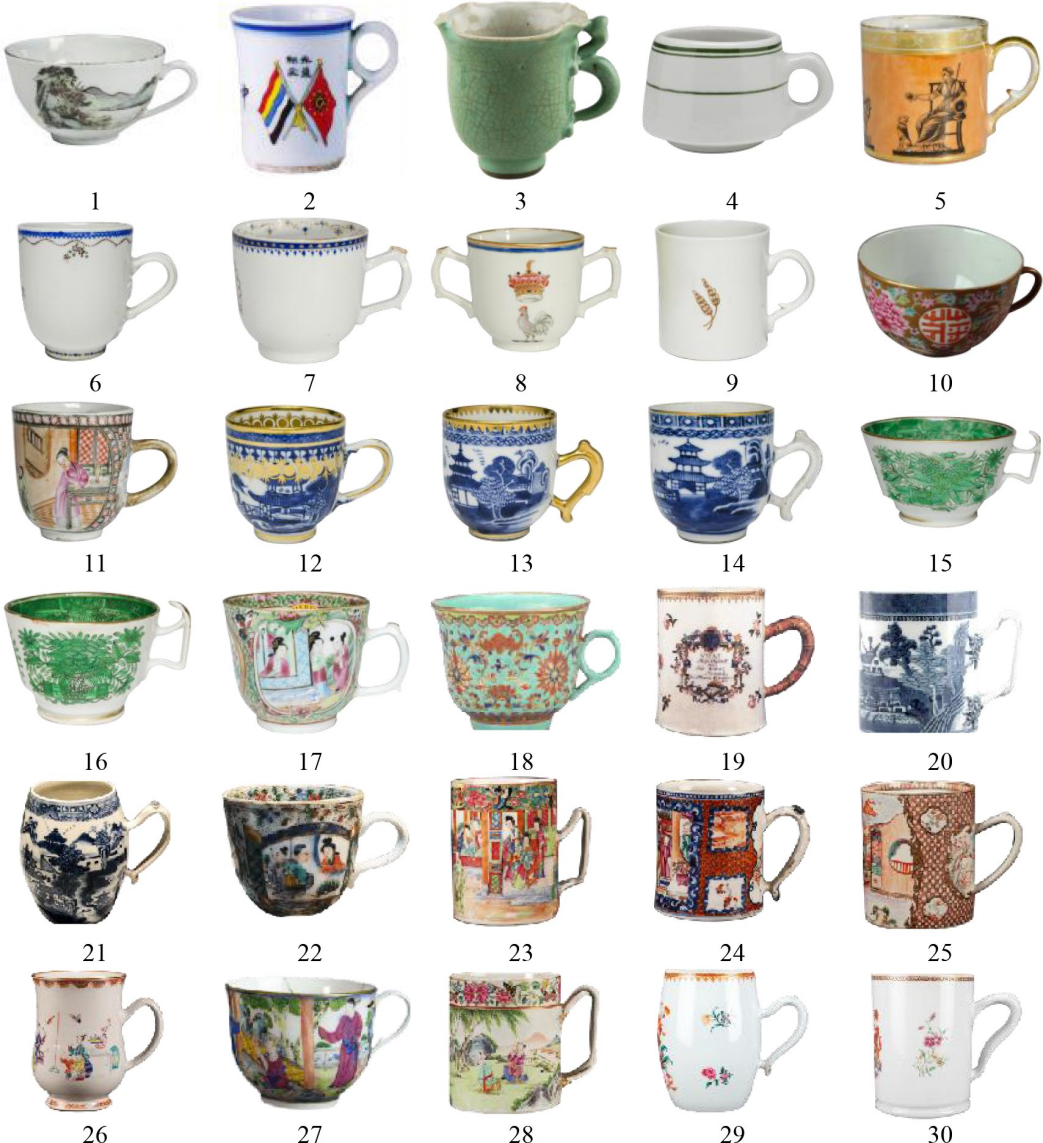
The 30 Lingnan mugs used for analysis in this paper.

This study primarily derives its data from a collection of 30 mugs **(Fig 1)** sourced from 13 museums, including the Zhejiang Province Museum, The Metropolitan Museum of Art, and the Brooklyn Museum. Among these, 20 samples are from the Qing Dynasty (1616–1912), and 10 from the Republic of China period (1912–1949), including 13 Canton mugs. These samples are coded as ’R’ and ’Q’ to represent the Republic of China and Qing Dynasty periods respectively, as detailed in **Table 1** (see S1 File).

**Table 1:** Sample data information sheet.

Code	Name	Collector	Period	Group
1	Republic of China period Famille Rose Porcelain Mug	Zhejiang Province Museum	Republic of China	R
2	Republic of China period Restoration Commemorative Mug	National Museum of China		
3	Qing Dynasty Green-glazed Mug with Gecko Handle and Flared Rim	The Metropolitan Museum of Art		
4	Vintage Trenle Blake China Co. Coffee Tea Mug	Philadelphia Museum of Art		
5	Mup from a Tea Service Showing Classical Motifs			
6	Mup for Export to the American Market			
7	Mup for Export to the American Market			
8	Cup with Double Handle			
9	Cup for Export to the American Market			
10	Republic of China Period Famille Rose Mug with "Jiang" Character and Floral Pattern	Henan Provincial Museum		
11	Canton Figure Mug	Brooklyn Museum	Qing Dynasty	Q
12	Chinese Export Coffee Mug, Blue & White			
13	18th Century Blue and White Mug			
14	Qing Qianlong Period Blue and White Porcelain Small Export Mug			
15	Cup, Chinese, for Export to the American Market, Hard-paste Porcelain with Enamel and Gilt Decoration	Philadelphia Museum of Art		
16	Cup, Chinese, for Export to the American Market, Hard-paste Porcelain with Enamel and Gilt Decoration			
17	Qing Dynasty Canton Figure and Floral Mug			
18	Green Ground Famille Rose Entwined Lotus and Bat Design Single-handled Mug	Chinese Palace Museum		
19	Famille Rose English Cobbler Illustrated Mug	The British museum		
20	Jingdezhen Kiln Blue and White Pavilion Illustrated Handled Cup	Shanghai Museum		
21	Blue and White Landscape Illustrated Mug	Jingdezhen Museum		
22	Canton Enamel Human and Floral Bird Pattern Teaware	The First Customs Museum of Guangdong		
23	Canton Enamel Floral, Butterfly, and Human Pattern Mug	Thirteen Factories Museum		
24	Canton Enamel Blue and White Brocade Ground Landscape and Character Illustrated Mug	Thirteen Factories Museum		
25	Canton Enamel Brocade Ground Ship and Character Illustrated Mug	Thirteen Factories Museum		
26	Ladies and Children Playing Pattern Mug	Thirteen Factories Museum		
27	Canton Enamel Courtyard Human Pattern Cup with Saucer	Thirteen Factories Museum		
28	Canton Enamel Floral, Butterfly, and Human Pattern Mug	Thirteen Factories Museum		
29	Canton Enamel Floral and Badge Pattern Barrel-shaped Mug	Guangdong Province Museum		
30	Canton Enamel Gilded Floral and Badge Pattern Mug	Guangdong Province Museum		

## 3 Results

### 3.1 Sample typing and coding

**Fig 2:**
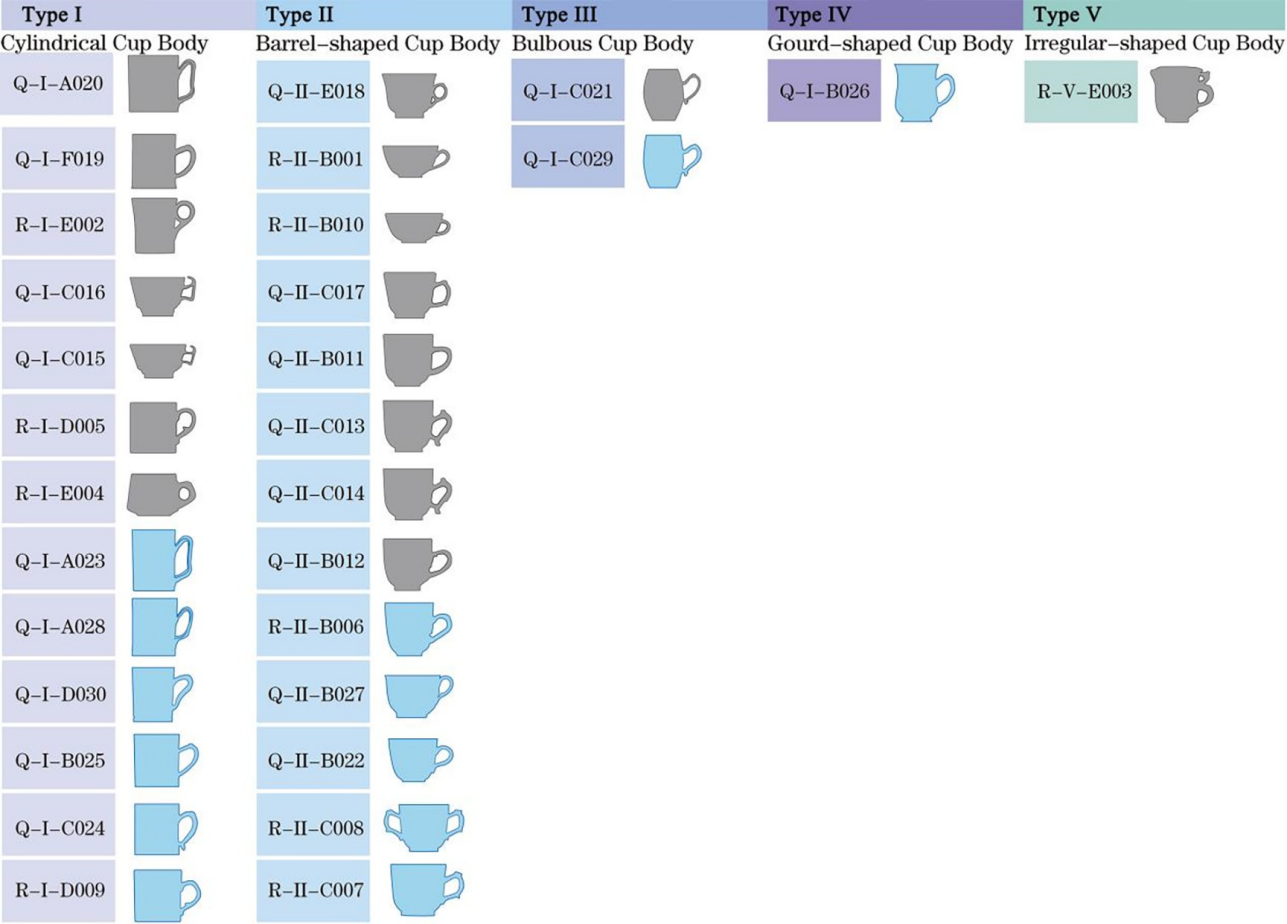
Mugs body and handle fractal chart.

In the second step, samples sharing similar characteristics are classified, and each category or element within a category is coded to identify several morphological variables for subsequent comparative analysis. According to **Table 1**, the mugs are divided into five types (**Fig 2)**: 1) Type I-Cylindrical mug body-直筒形 (sample-30), characterized by a vertical alignment of the cup mouth to the body, with the mouth diameter equal to the base; 2) Type II-Barrel-shaped mug body-桶形 (sample-29), where the body is barrel-shaped; 3) Type III-Bulbous mug body-鼓腹形, with a mouth diameter greater than the base diameter, and the lower body contour outwardly bulged; 4) Type IV-Gourd-shaped mug body-葫芦形 (sample-26), where the body is gourd-shaped with a continuous double-curve contour; 5) Type V-Irregular-shaped mug body-异形, noted for its irregular shape.

**Fig 3:**
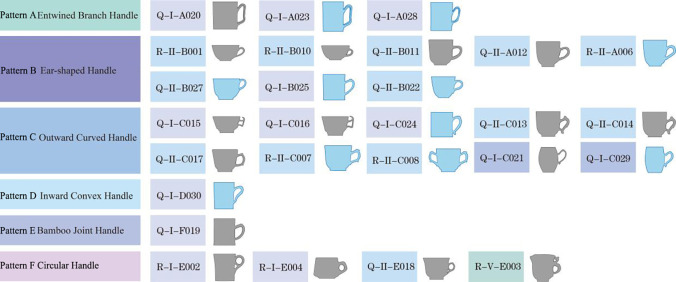
Morphological and handle combination typing table of the samples.

Based on the initial coding in **Table 1**, further coding of the 30 mugs integrates dynasty, types, patterns, and codes. For example, sample 23 is coded as B’aI-xx type. **Fig 3** illustrates a typology of morphology and handle combinations. The handles are subdivided into six forms: 1) Pattern A-Entwined Branch Handle (缠枝状-◆), featuring a morphology resembling intertwined branches, influenced by ancient plant decorative motifs, reflecting a traditional appreciation of nature; 2) Pattern B-Ear-shaped Handle (耳廓状-○), shaped akin to a human ear, potentially related to contemporary social customs and bodily aesthetics; 3) Pattern C-Outward Curved Handle (外翘状-▽), a dynamic, undulating design enhancing the artistic expression of the mugs; 4) Pattern D-Inward Convex Handle (内凸状-◇), showcasing a concave shape that offers a more stable grip; 5) Pattern E-Bamboo Joint Handle (竹节状-●), mimicking the natural characteristics of bamboo, embodying resilience and elegance central to Eastern philosophy; 6) Pattern F-Circular Handle (环状-△), exhibiting a circular morphology that echoes ancient aesthetics of connection and completeness.

**Fig 4:**
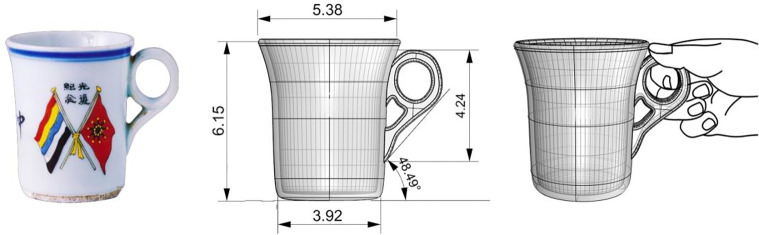
Analysis of mug‘s scale and hand grip posture—1:1 model reduction with sample 2.

The morphology of Qing Dynasty mugs primarily features Type A and Type C, followed by Type B and Type D, with Type A mugs being predominant. For instance, sample 30 **(Fig 4)** has a Type A body; its handle design aligns with the ergonomic support points for a single-hand grip, matching the positions of the thumb, index, and middle fingers. Through 1:1 three-dimensional model reconstruction and data measurement, its capacity is deemed moderate, indicating that the weight of the liquid it carries is manageable with one hand. Conversely, Sample 3’s capacity approaches that of a modern mug, typically around 500ml, meeting most contemporary needs for mug portability and use. This reflects the uniform specifications of military enamel mugs during the Republic of China period due to wartime influences. Compared to sample 2, Sample 30 exhibits finer detail in aspects like the rim, thickness, handle junction, and base curvature. The thickness relates closely to the density, temperature, and craftsmanship of porcelain production, indicating that Qing Qianlong period mugs benefited from advanced porcelain manufacturing techniques and conditions.

### 3.2 Curve extraction and typing

**Fig 5:**
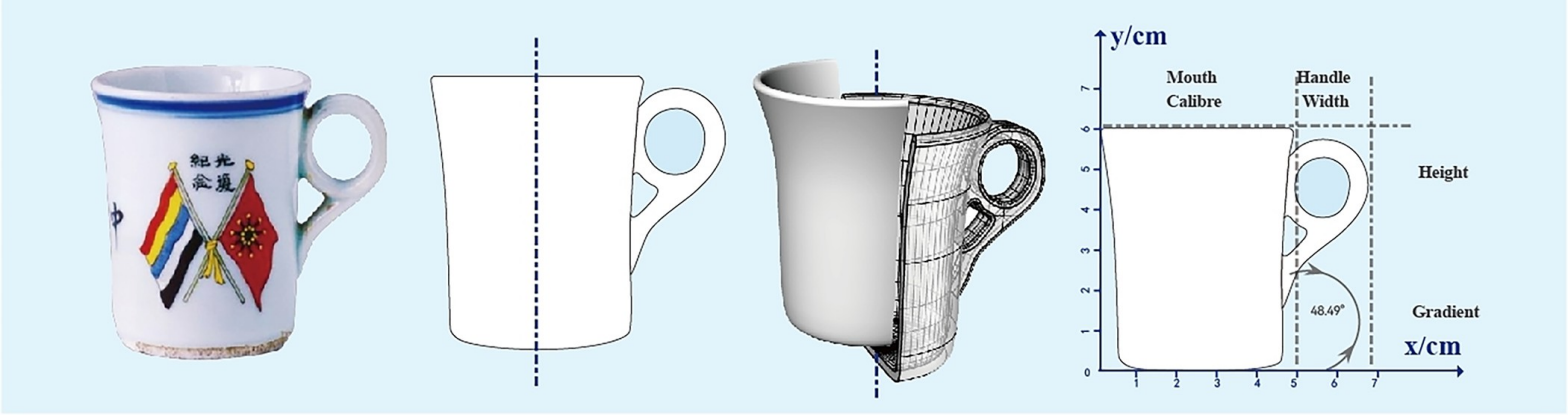
Step-by-step diagram of quantitative typological analysis.

In the next phase, spline curves in Adobe Illustrator are utilized to outline the silhouettes of all samples listed in **Table 1**, converting the edge curves into a series of data points for localization within a graphical coordinate system. Subsequently, axes are drawn for each coded graphic, and an in-depth analysis of localized features with significant morphological changes is conducted to compare the self-similarity in the evolution of forms. For samples with missing data, proportional three-dimensional modeling is performed in Rhino based on the available data, and measurements are taken to collect the required data, as depicted in **Fig 5**, which illustrates the basic steps of quantitative typological analysis.

### 3.3 Data measurement

**Fig 6:**
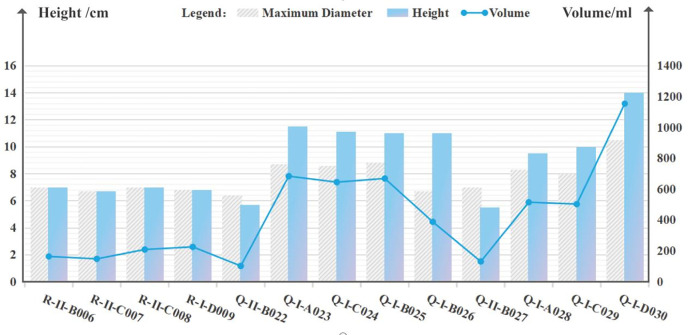
Scale trends of Canton mugs in 18th-20th centuries.

Next, phenograms for all mugs are plotted on a unified set of axes **(Fig 6)** to provide a quantified description of morphological changes. The volume (V) of the mugs can be calculated using **Eq I**, and for samples with an inclination (I) less than 90°, it is calculated using **Eq II**.


$$V=\pi r^{2}\;h$$ Eq I

In **Eq I**, V represents volume; π represents pi, valued at 3.14; r represents the radius of the mug’s base; h represents the height of the mug.


$$V=1/3\pi h(r^{2}+R^{2}+rR)$$ Eq II

In **Eq II**, V represents volume; π represents pi, also valued at 3.14; r represents the radius at the top of the mug; R represents the radius at the bottom of the mug; h represents the height along the side of the mug measured at the inclination.

**Fig 6** illustrates the scale variations of Canton mugs, while **Table 2** presents detailed data on their maximum diameter (MD), height (H), inclination (I), volume (V), and the dimensions of the bottom and handle. Specifically, the Handle-side Junction Angle and Wall-side Junction Angle denote the angular measurement (∠) between the handle and the cup wall, as well as the height of the junction (y) and its offset from the central axis (x).

**Table 2:** Morphological data relating to Canton mugs in the 18th-20th centuries.

Dimensional data														
Types	Sample			Maximum Diameter (MD)/ cm				Height (H)/cm		Inclination (I)/°				Volume(V)/ml
R-II-B006	6			7				7		50°				163
R-II-C007	7			6.7				6.7		51°				147
R-II-C008	8			7				7		51°				208
R-I-D009	9			6.8				6.8		90°				225
Q-II-B022	22			6.4				5.7		48°				101
Q-I-A023	23			8.7				11.5		90°				683
Q-I-C024	24			8.6				11.1		90°				644
Q-I-B025	25			8.8				11		90°				668
Q-I-B026	26			6.7				11		53°				387
Q-II-B027	27			7				5.5		46°				130
Q-I-A028	28			8.3				9.5		90°				514
Q-I-C029	29			8				10		73°				502
Q-I-D030	30			10.5				14		89°				1153
**Bottom and handle size data**														
Types			**Bottom Sizes**						**Handle Sizes**					
			**Height (H)/cm**			**Diameter (D**			**Wide (W)/cm**			**Height (H)/cm**		
R-II-B006			7			7			2.5			4.3		
R-II-C007			6.7			6.7			2.6			4.2		
R-II-C008			7			7			2.3			4.6		
R-I-D009			6.8			6.8			2.6			4.5		
Q-II-B022			5.7			2.9			2.5			3.8		
Q-I-A023			11.5			8.7			3.5			9.2		
Q-I-C024			11.1			8.6			4.2			8.5		
Q-I-B025			11			8.8			3.9			7.6		
Q-I-B026			11			4.9			3.5			6.4		
Q-II-B027			5.5			3.8			2.3			3.6		
Q-I-A028			9.5			8.3			2.9			7.5		
Q-I-C029			10			6.5			3.5			6		
Q-I-D030			14			7.5			4.9			9.1		
**The angle of the tangent between the handle and the mug wall (∠), the height of the tangent point (y) and its offset from the center axis (x)**														
Types		Handle-side Junction Angle						Wall-side Junction Angle						
		**∠**			Y/cm		X/cm	**∠**			Y/cm		X/cm	
R-II-B006		43°			2.7		4.4	50°			3.5		3.5	
R-II-C007		38°			2.2		4.5	51°			3.35		3.3	
R-II-C008		39°			2.6		4.6	51°			3.5		3.5	
R-I-D009		47°			2.6		5.1	90°			3.4		3.4	
Q-II-B022		36°			1.8		3.4	72°			2		2.2	
Q-I-A023		34°			2.6		5.4	88°			2.1		4.4	
Q-I-C024		48°			3.1		6	90°			0		4.3	
Q-I-B025		39°			4		6.4	90°			0		4.4	
Q-I-B026		34°			2.7		3.9	50°			2.3		2.7	
Q-II-B027		44°			1.6		2.9	68°			1.7		2.3	
Q-I-A028		34°			3.3		5.3	90°			0		4.1	
Q-I-C029		38°			3.6		4.1	73°			3.7		2.9	
Q-I-D030		53°			3.5		5.2	88°			2.1		3.8	
Average		40°			2.7		4.7	73°			2.1		3.4	
Middle value		39°			2.7		4.6	73			2.1		3.5	

### 3.4 Analytical mapping

**Fig 7:**
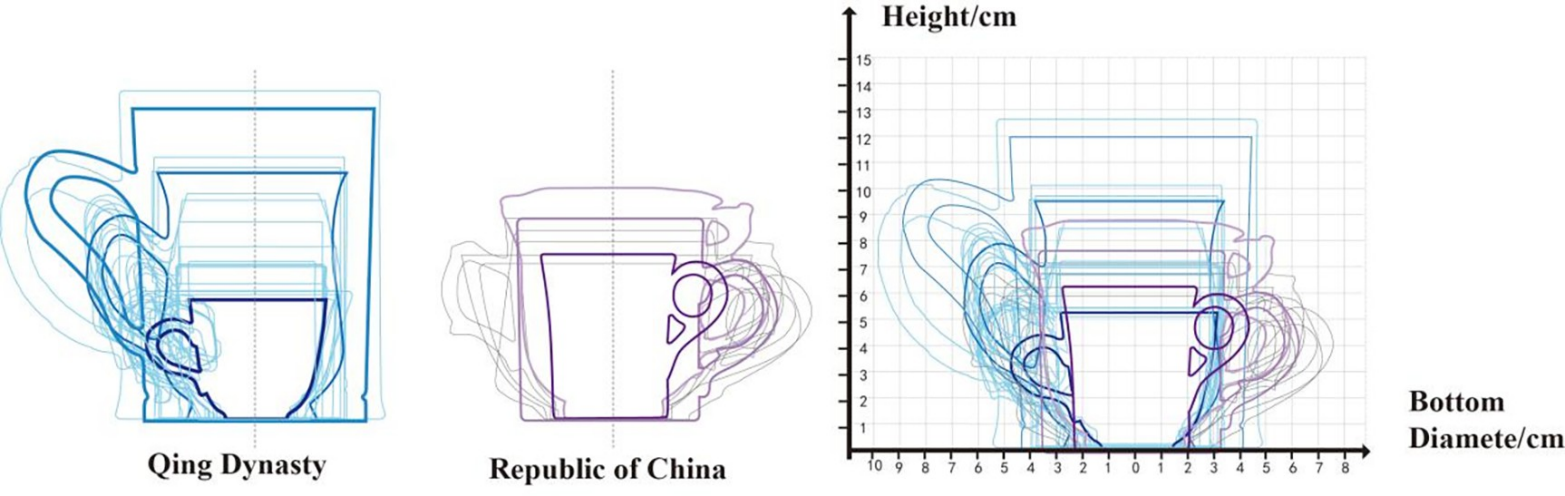
Changes of the bottom morphologies.

**Fig 8:**
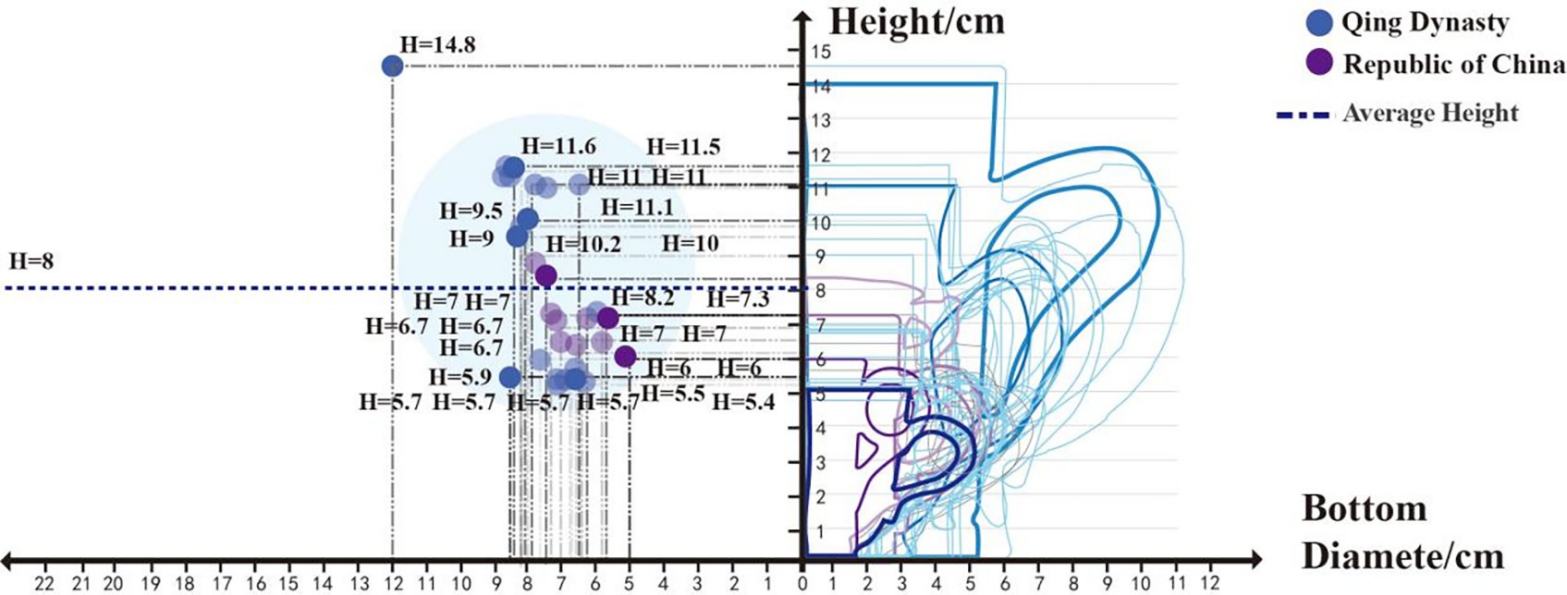
Changes of the handle morphologies.

**Fig 9:**
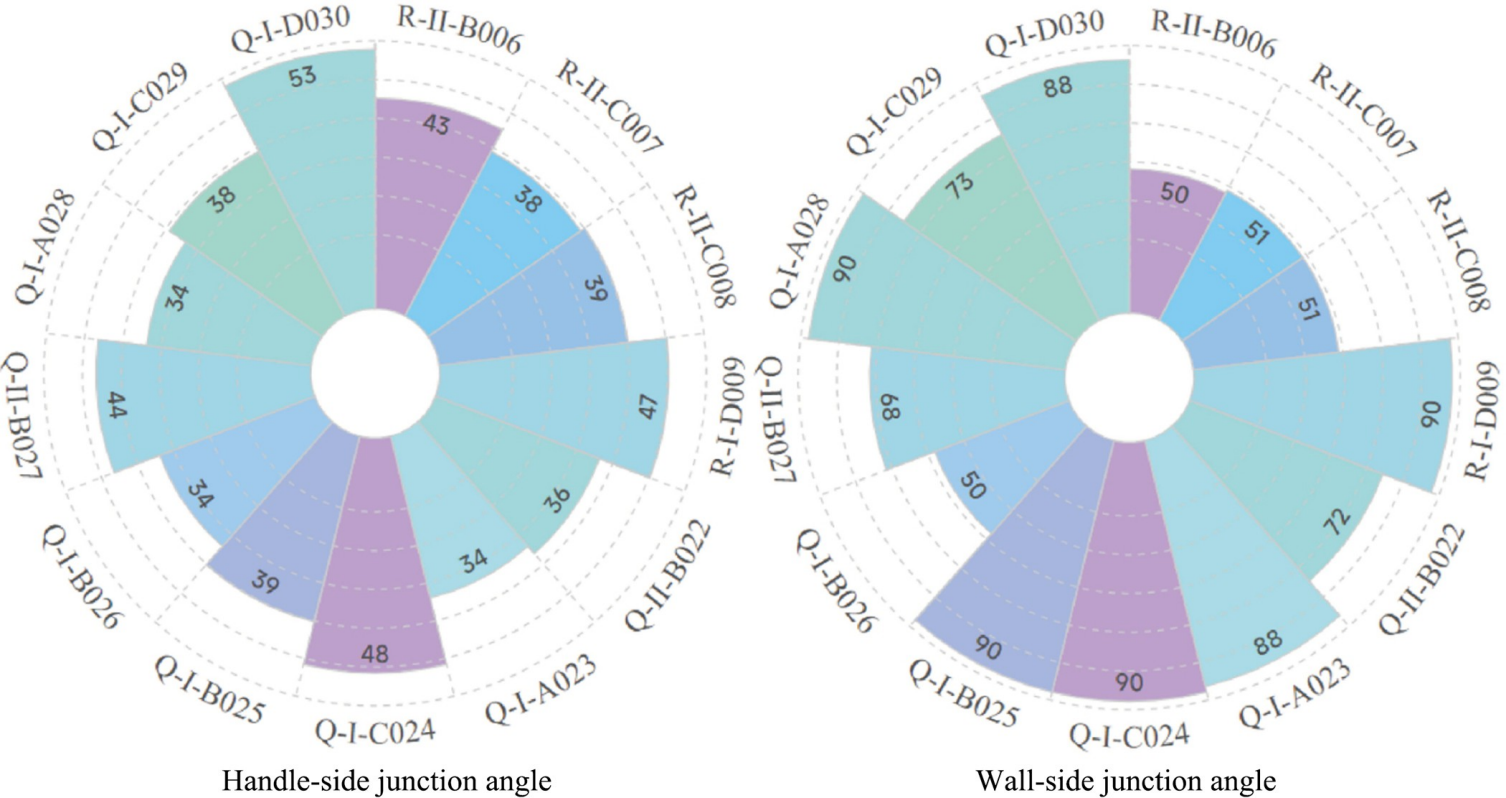
Variation of Handle-side junction angle and Wall-side junction angle for mugs.

Based on the data from **Table 2**, this paper assigns uniform coordinates to these samples to compare the morphological changes of the objects. **Figs 7 and 8** compares the morphology of the bases and handles of Canton mugs. **Fig 9**, derived from **Table 2**, contrasts the changes in the Handle-side Junction Angle, which is the angle formed by the outer side of the handle and the horizontal line of the mug base, and the Wall-side Junction Angle, which is the angle between the mug’s inclination and the horizontal line of the base.

The range for the Handle-side junction angle of thirteen Canton mugs is 34°-53°. These minor variations are likely related to multiple factors, including but not limited to cup-making techniques, aesthetic preferences, functionality, production models, technological advancements, and shifts in market demand. In contrast, the Wall-side junction angles vary more significantly (51°-90°), possibly designed for enhanced stability between the handle and the mug body [19], and also align with ergonomic principles. The size of these angles relates to the stability of the mugs, with larger angles reducing the likelihood of the mugs tipping over [20].

## 4 Discussions

### 4.1 Characteristics of the stylized classification of mugs

**Table 3** categorizes the design features of thirty mugs. The sample cups are predominantly characterized by a bulbous cup body and a tail outward curved handle. Type c-III combines features of tail and top outward curves. Type c-V features an inwardly convex interior handle contour. Within the same group, the interior morphology of Type C-III’s handle forms a convex closure with the cup wall, thus classified as an inward convex handle. Type F-V represents a combination of an irregular-shaped body and a top outward curved handle, with the handle’s exterior contour forming an interlocking ring shape.

**Table 3:** Classification basis for stylistic features of 30 mugs.

	Bamboo Joint Handle	Entwined Branch Handle	Outward Curved Handle	Circular Handle	Ear-shaped Handle	Inward Convex Handle
I-Cylindrical	E-I	A-I	C-I	-	B-I	-
II-Barrel-shaped	-	-	C-II	-	-	-
III-Bulbous Cup Body	E-III	-	C-III C-III	F-III F-III	B-III	D-III D-III
IV-Gourd-shaped	-	-	C-III	-	B-IV	-
VIrregular-shaped	E-V			F-III		

### 4.2 Patterns of change in the morphology of mugs

**Fig 10:**
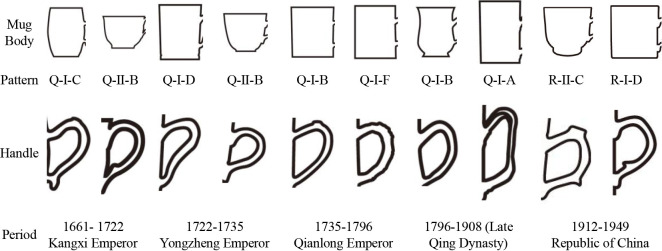
The changing patterns of body and handle morphologies of mugs.

**Fig 10** illustrates the morphological evolution process of Lingnan mugs. The progression from Arc Barrel→Bulbous Cup Body→Cylindrical Cup Body, marks the developmental stages of Lingnan mugs’ morphology from the Qing dynasty to the Republic of China period. Similarly, the handle morphology has also undergone complex transformations from outward curved morphology, to ear-shaped morphology, and finally to entwined branch morphology.

The insulating qualities and stability offered by the arc-barrel and bulbous cup body types [19] catered to the ancient Chinese demands for tea-drinking practices. The emergence of cylindrical cup bodies in later periods can be attributed to a shift towards utilitarian and minimalist design preferences. The design of outward-curved handles enhances grip stability and comfort, while ear-shaped handles add decorative elements and signify social status. The gradual evolution towards entwined branch handle morphology may reflect the influence of East-West cultural exchanges, showcasing the Qing dynasty’s adaptive integration in its open and interactive cultural policies.

### 4.3 Volume adjustment and morphological changes in mug capacity under changing drinking demand

**Fig 11:**
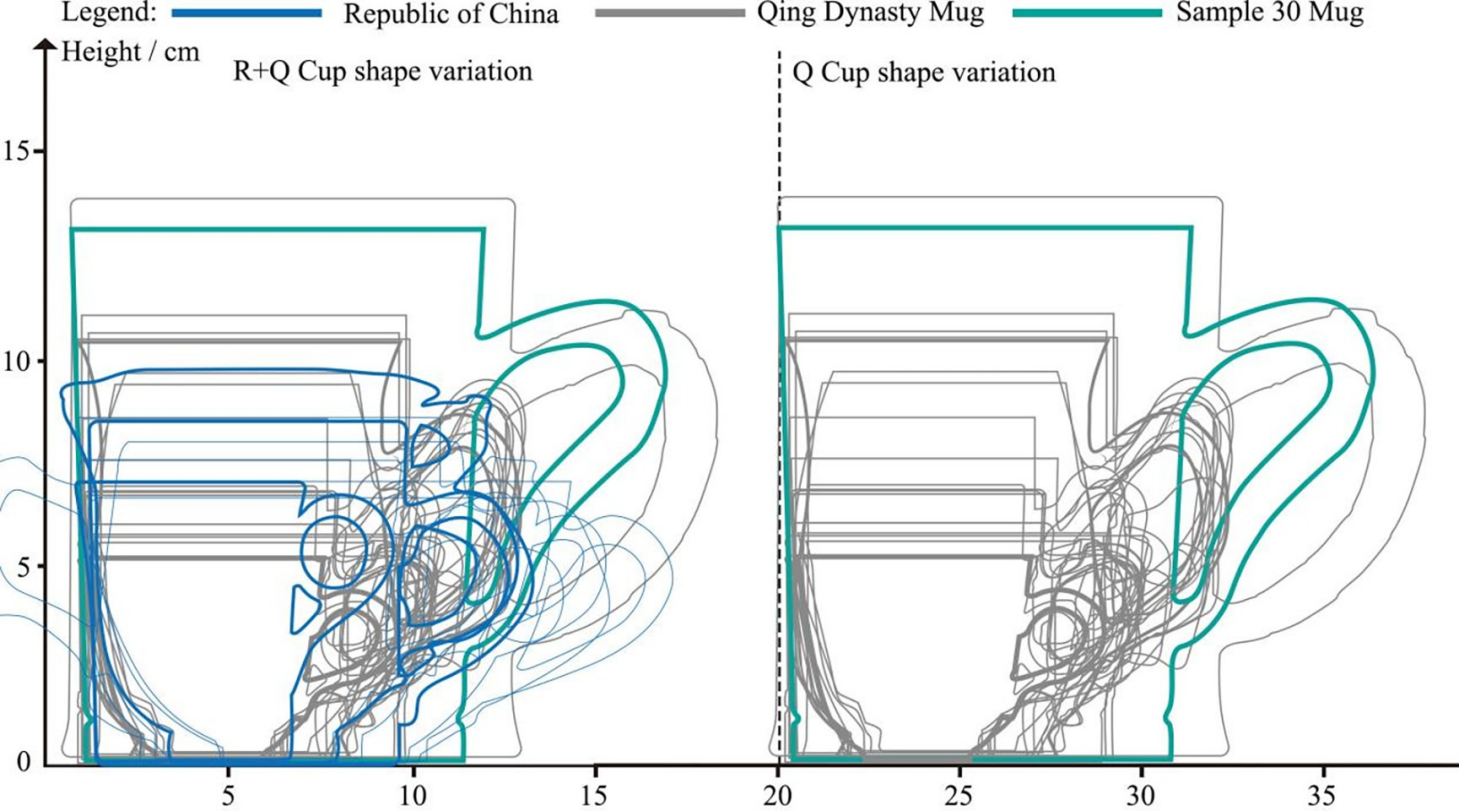
Changes in overall morphology in 30 mugs.

During the Qing dynasty’s Qianlong period, the standardization of mug dimensions, including height, rim diameter, and handle design, was evident **(Fig 11)**. There was a notable variation in the capacity of Qing dynasty mugs, categorized into large (>500ml), medium (200-500ml), and small (<200ml) sizes, correlating with the diverse beverage consumption needs such as coffee and beer. The average capacity was 423ml, with diameters and heights at 7.6cm and 8.5cm, respectively. The significant capacity range, with a maximum difference of 1588ml between samples 19 and 2, reflects shifts in drinking habits. This period marked the coexistence of traditional tea consumption with emerging beverages like coffee and beer. Although small-capacity mugs played a transitional role, they constituted a minor proportion of the total samples, indicating a predominant consumer preference for medium and large capacities. Moreover, the capacity of mugs during the Republic of China era closely mirrored those of the Qing dynasty. Due to their durability and portable designs, enamel mugs were extensively utilized during wartime, aligning more with modern mass production practices due to their uniformity and cost-effectiveness.

### 4.4 Social factors influencing the morphologies of mugs

The evolution of Chinese cups from the Zhou dynasty’s religious ceremonial utensils to the widespread use of the Han dynasty celadon illustrates a fusion of functionality and symbolism [32]. The porcelain craftsmanship of the Zhou dynasty, closely integrated with ritualistic activities, propelled a diversification in design [33]. As the use of bronze ware increased during the Spring and Autumn and Warring States periods, cups displayed more complex artistic forms and social identifiers [34]. The standardization of weights and measures during the Qin dynasty, coupled with stringent craftsmanship requirements, refined the production scales of artifacts [35]. The widespread adoption of celadon in the Han dynasty not only reduced production costs but also enriched the cups with symbols of longevity and health [36]. During the Tang and Song periods, as the pursuit of craftsmanship aesthetics intensified, cup designs evolved to harmonize practicality with aesthetics, exemplified by the innovations of Ding ware and Ge ware [37]. The advent of industrialization and the rise of foreign trade simplified the functions and morphology of cups, while customized production fostered diversity in forms, marking the transition of Chinese porcelain cups from traditional handicrafts to modern industrial production and highlighting the profound impacts of globalization on local crafts [38].

From the Yuan dynasty through to the establishment of new China, each era’s mug morphology was shaped by contemporary socio-cultural influences and, in turn, impacted the designs of subsequent dynasties **(Table 4)**. Yuan dynasty mugs were robust [39], Ming dynasty adaptations incorporated refined traditional Chinese elements like blue-and-white porcelain [40], and Qing dynasty designs, while initially retaining Ming styles, gradually integrated more Western elements like underglaze color [41]. During the Republic of China period, designs were further modernized, reflecting openness and modernization trends [42]. The acceptance and use of mugs, a form common in the West, gradually permeated Chinese regions against the backdrop of cultural integration. Post-revolutionary China then saw mug designs that prioritized simplicity and utility, reflecting new societal needs and aesthetic preferences.

**Table 4:** Morphology evolution of the mug and its influencing factors.

Dynasty	Year	Events	morphology feature
Yuan	1271–1368	The design of mugs was significantly influenced by the Mongol Empire’s extensive cultural exchanges, drawing on design elements from Central Asian and Persian styles through trade on the Silk Road. During this period, mugs exhibited a rugged and utilitarian style, reflecting the Yuan dynasty’s multicultural society and its emphasis on pragmatism.	Incorporating Central Asian and Persian styles, it focuses on practicality, but with rugged styling.
Ming	1368–1616	As this era focused on traditional culture, the design of mugs not only continued the styles from the Yuan dynasty but also incorporated more refined Chinese traditional elements, such as the designs of blue and white porcelain cups. The literati’s reverence for tea culture during this period was also reflected in the intricate designs of the mugs.	Sophisticated design influenced by tea culture; using more traditional Chinese elements.
Qing	1616–1912	With the cultural integration brought about by maritime trade between the East and the West, the mugs of this era were influenced by both the Manchu and European cultures. As customized products, the mugs displayed a diversity of features, such as underglaze painting and Kwon-Glazed techniques, demonstrating the Qing dynasty’s trend towards openness and cultural integration.	Mug presents a diversified design that is a fusion of Chinese and Western art, and shows the development of the trend of simplicity.
Republic of China	1912–1949	The development of industrial production techniques led to a modernization trend in mug design, establishing the basic morphology of the cylindrical cup body-type, with a focus on aesthetics, practicality, and ergonomic handling. Additionally, as mugs increasingly became everyday drinking vessels, there was a trend toward decreasing size.	The form of the mug is dominated by the Cylindrical mug body and focuses on practicality and ergonomics.
After New China	After 1949	Innovations in mass production technology standardized mug production, ensuring consistency in morphology.	Morphology is parsimonious and the body types converge.

This research clarifies the evolution of mug morphologies from the Qing Dynasty through the Republican era in China, influenced profoundly by international trade and cultural exchanges. This progression has seen a shift from traditional forms to designs that are both more practical and aesthetically diverse. From a social perspective, these mugs were not only widely circulated as everyday items but increasingly came to symbolize personal identity and social status. The push towards economic globalization, particularly in trade between China and Western nations, has fostered a blending of cultural elements in mug designs, meeting consumers’ dual demands for functionality and aesthetic appeal. The incorporation of Western design styles into Lingnan mugs is evident with the progression of cultural integration.

### 4.3 Research value

In contrast to a focus purely on functionality, the societal influences behind material morphology changes have received less attention [43]. This is particularly true in the analysis of traditional Chinese porcelain, even though Guo [44] emphasized this aspect in his study of excavated bowls. However, his research remained focused on material aspects rather than cultural implications. Considering Lingnan’s commercial culture, this paper examines the social drivers behind morphological changes in mugs designed for export to Europe. Utilizing quantitative typological analysis and supplemented by historical documentation, this study measures the morphology of 30 mugs to reveal the impacts of technological innovation, cultural exchange, and social transformation on their evolutionary patterns.

This paper systematically analyzes the diversity in the body and handles morphologies of mugs from the Qing Dynasty to the Republican period, enhancing the quantification of vessel morphology through quantitative morphological type analysis. The findings closely align with Kharchenkova’s [45] research on the popularization trends of mugs and resonate with the detailed classifications and evolutionary analyses of ancient porcelain morphologies by Liu and Zhang [46] and Zheng [47]. Further discussions confirm that the morphology of these objects results from a confluence of functional, cultural, and social influences, echoing Yang’s [48] conclusions about the morphology of pedestal cups. Additionally, this study supports the perspectives of Dong [49], Huang [50], and Zhang and Han [51] on how societal, cultural, and political factors influence the morphology of exported vessels, while also emphasizing the significance of aesthetics, cultural customs, and ergonomics in the design of mug handles, complementing Feng’s [52] principle of functionality first. Overall, this research offers a comprehensive, detailed, and profound theoretical analysis of ancient mug morphologies, providing both academic value and practical insights for product design.

The study acknowledges limitations in data collection and cultural diversity, constrained by museum display conditions such as inadequate image angles and resolution details, leading to biases in modeling and computation. Compared to Zhang [53], this study does not fully cover the religious and philosophical thoughts of the corresponding period, possibly overlooking some cultural influences. Additionally, unlike Feng [52], it does not delve deeply into the relationship between vessel morphology and the specifics of hand size and grip habits. To address these limitations, the paper proposes improvements: 1) adopting advanced digital imaging technologies like 3D scanning and augmented reality to capture more comprehensive and precise images of vessels, thereby enhancing the quality and detail accuracy of data collection; 2) expanding the research framework to incorporate interdisciplinary perspectives such as anthropology, sociology, and ergonomics to explore the cultural symbolism and ergonomic functionality of vessels more thoroughly.

## 5 Conclusions

Analysis of 30 mugs from the Qing Dynasty to the Republic of China period reveals a classification into five types and six patterns, with cylindrical bodies and ear-shaped handles being most common during the Qing era. The morphological evolution of the mug bodies from arc-barrel to bulbous to cylindrical reflects a shift from initial thermal insulation functions to practical and economical consumer preferences. Additionally, the diversity in handle designs also illustrates varying characteristics. Comparatively, the mugs from the Republic of China period resemble those produced by modern industrial methods more closely. In terms of capacity, these mugs exhibit significant variations, with an average capacity of 356 ml, ranging from a minimum of 85 ml to a maximum of 1673 ml, indicating a wide disparity that reflects diverse consumer preferences and social drinking cultures. The analysis of the handle-side junction angles of Canton mugs shows concentration, whereas the wall-side junction angles vary more significantly, correlating with functional demands and stability considerations.

To comprehensively understand the design of export mugs from the Qing period, one must consider their practicality, aesthetic values, and their role within the socioeconomic context. It is also crucial to examine how these artifacts reflect social hierarchies, economic conditions, and shifts in global trade patterns. This paper discusses how social factors influence the morphology of material culture, providing a theoretical framework to explain the social drivers behind morphological changes in cultural items. Future research will continue to focus on the cross-regional cultural impacts and dissemination of export artifacts, revealing the dynamics of cultural exchange and material culture under globalization.

## Supporting information

S1 File Morphological data on Lingnan export mugs. (ZIP)
